# Motivational Regulations Across the Stages of Change for Exercise in the General Population of Monterrey (Mexico)

**DOI:** 10.3389/fpsyg.2018.02368

**Published:** 2018-12-03

**Authors:** Jorge Zamarripa, Isabel Castillo, Raúl Baños, Maritza Delgado, Octavio Álvarez

**Affiliations:** ^1^Faculty of Sports Organization, Universidad Autónoma de Nuevo León, San Nicolás de los Garza,Mexico; ^2^Faculty of Psychology, Universitat de València, Valencia, Spain; ^3^Faculty of Sports, Universidad Autónoma de Baja California, Mexicali, Mexico; ^4^Faculty of Psychology, Universidad Autónoma de Nuevo León, San Nicolás de los Garza, Mexico

**Keywords:** motivation, self-regulation, adherence, exercise, BREQ-3, stages of change

## Abstract

Few studies have examined exercise adherence in the Mexican population using self-determination theory proposals and the stages of change model. The objectives of this study were:(a) to translate and adapt the Behavioral Regulation in Exercise Questionnaire-3 (BREQ-3) to Mexican Spanish and examine its internal consistency and factorial structure (six dimensions); and (b) to analyse variations in behavioral regulations using the stages of change model. This study included 530 participants between 11 and 76 years old who lived in the metropolitan area of the city of Monterrey, Nuevo Leon, Mexico. The Mexican version of the BREQ-3 presented an acceptable six-factor model that agrees with the theory and has good internal consistency. Results showed that the less self-determined regulations (i.e., external and amotivation) predominated in the first stages of change (i.e., pre-contemplation and contemplation) and decreased in the last stages (i.e., action and maintenance); by contrast, the more self-determined regulations (i.e., intrinsic, integrated, and identified) predominated in the last stages (i.e., action and maintenance) and were lower in the first stages (i.e., pre-contemplation and contemplation). Linking these two theoretical constructs contributes to understanding physical exercise adherence.

## Introduction

Being physically inactive is a major health risk factor that has been well-documented in the literature. Despite the health benefits associated with physical activity, in the Mexican context almost three quarters of people over 19 years old are overweight or obese (National Institute of Public Health, 2016). Although many people know the benefits of being physically active, not everyone starts or continues an exercise program (Berger et al., 2002), and so more research is needed to better understand physical exercise adherence. Previous research has shown that the motivation underlying exercise behavior could partly explain why some individuals initiate or drop-out from exercise programs (Thogersen-Ntoumani and Ntoumanis, 2006).

Self-determination theory (SDT; Deci and Ryan, 1985, 2002) is one of the most widely studied theoretical constructs in recent years in different contexts (Vallerand et al., 2008), including physical exercise contexts. This theory suggests that individuals can be involved in an activity for different reasons or motives, and these reasons can be detected as motivational regulations that are more or less self-determined (autonomous). Deci and Ryan (1985) proposed that six different forms of regulation can be situated along a self-determination continuum, with behavior ranging from high levels of autonomy (*intrinsic motivation*), to medium levels (*extrinsic motivation*), and reaching low levels (*amotivation*). Specifically, these behavioral regulations are labeled intrinsic regulation, integrated regulation, identified regulation, introjected regulation, external regulation, and amotivation.

*Intrinsic motivation* is present when an individual performs an activity merely for the pleasure of experiencing it, without expecting a reward or trying to avoid punishment. On the other end of the continuum lies *amotivation*, where the person does not have any motivation to engage in an activity (Deci and Ryan, 2000). Individuals who are not motivated to practice sports do not seek social, affective, or material objectives; instead, they experience negative sensations such as incompetence, apathy, and even depression (Vallerand, 2001). *Extrinsic motivation* is found between the extremes of intrinsic motivation and amotivation, and it refers to performing an activity with the aim of obtaining a separable result (Ryan and Deci, 2000).

Within SDT, a mini-theory called organismic integration theory (Deci and Ryan, 1985) was introduced to explain the different forms of extrinsic motivation and the contextual factors that promote internalization and the integration of behavioral regulation. This theory proposes an order or sequence for the degree of autonomy with which extrinsic motivation is regulated. The sequence consists of integrated, identified, introjected, and external regulation. *External regulation* is the least autonomous type of extrinsic motivation, and it includes the classic case of motivation to obtain rewards or avoid punishment. *Introjected regulation* is somewhat more internalized and based on behaviors carried out to avoid guilt, anxiety, and shame, or to improve the ego, feelings of value, or pride. *Identified regulation* involves awarding a conscious value to a behavior in such a way that the action is accepted when it is personally important (Ryan and Deci, 2000). Lastly, *integrated regulation* represents the most autonomous form of extrinsic motivation (Ryan and Deci, 2000). Integration occurs when regulations have been identified, evaluated, and assimilated.

One of the instruments most frequently used to measure the different forms of behavioral regulation in exercise contexts is the Behavioral Regulation in Exercise Questionnaire (BREQ; Mullan et al., 1997). The BREQ was composed of 15 items distributed in four factors: external regulation (four items), introjected regulation (three items), identified regulation (four items), and intrinsic motivation (four items). The authors did not include the integrated regulation subscale, and the items that tapped amotivation were dropped due to high levels of skewness. The results of confirmatory factorial analyses have supported the factorial validity of the instrument in different areas (Mullan and Markland, 1997; Ingledew et al., 2004; Landry and Solmon, 2004), highlighting the behavior of university students who practice a sport (Wilson et al., 2002).

Later, Markland and Tobin (2004) reincorporated the amotivation items in a second version of the instrument, called the BREQ-2. This version contains 18 items divided into five dimensions: amotivation (four items), external regulation (four items), introjected regulation (three items), identified regulation (three items), and intrinsic motivation (four items). The authors did not include the integrated regulation subscale because they found that it was not possible to empirically distinguish between integrated and identified regulation, and, due to an error, they omitted one identified regulation item. Previous research has supported the BREQ-2's factor structure and subscale reliability in different languages (e.g., Moreno et al., 2007; Moustaka et al., 2010; Costa et al., 2018).

To address the limitations of the BREQ-2, an integration subscale and a new additional introjected item were included to produce the BREQ-3 (Markland and Tobin, 2004; Wilson et al., 2006). This new version contains 23 items divided into six subscales: amotivation (four items), external regulation (four items), introjected regulation (four items), identified regulation (three items), integrated regulation (four items), and intrinsic motivation (four items). The BREQ-3 obtained acceptable fit indexes and adequate reliability in different languages (Wilson et al., 2006; González-Cutre et al., 2010; Guedes and Sofiati, 2015; Cid et al., 2018; Costa et al., 2018). Furthermore, several researchers have provided additional support for the BREQ-3 demonstrating that scores on the questionnaire discriminated among male and female groups (e.g., González-Cutre et al., 2010; Guedes and Sofiati, 2015; Su et al., 2015; Cid et al., 2018).

The different forms of motivational regulation directly predict a wide variety of cognitive, affective, and behavioral outcomes (Vallerand, 1997). Specifically, self-determined regulations (i.e., intrinsic and identified regulations) are related to more adaptive outcomes, compared to less self-determined regulations (i.e., introjected and external regulation) or amotivation (e.g., Daley and Duda, 2006; Thogersen-Ntoumani and Ntoumanis, 2006; Lewis and Sutton, 2011).

Several studies have examined adherence to exercise through the union of two theoretical constructs, the self-determination continuum and the stages of behavioral change proposed by the transtheoretical model (Prochaska and Di Clemente, 1982). This model supports the perspective that physical exercise is a dynamic behavior, and that people pass through five *stages* of behaviors in their attempts to change their sedentary lifestyle into one that is physically more active. These stages include the pre-contemplation stage (a stage where the subject is physically inactive and has no intention of changing), the contemplation stage (the subject is inactive but has the intention to change), the preparation stage (the subject is active but does not carry out the recommendations for healthy practice), the action stage (the subject is active and complies with the recommendations for healthy practice, but has not completed six full months of regular practice), and the maintenance stage (stage where the person has performed healthy physical activity for more than 6 months). Individuals can move through the different stages (in both directions) as they perceive success in changing or maintaining their lifestyles (Prochaska et al., 1992b).

Mullan and Markland (1997) explored the relationship between behavioral regulations of exercise behavior and stages of change for exercise. They found that people in the early stages of change were less self-determined in their behavioral regulation than those in the later stages. Similar results were found by Daley and Duda (2006), who showed that participants in the early stages were less self-determined in the regulation of their exercise than those in the later stages of change. Thogersen-Ntoumani and Ntoumanis (2006) found that exercisers in the maintenance stage of change displayed significantly more self-determined motivation to exercise than those in the preparation and action stages. Permanence in a specific stage has repercussions in terms of the type of intervention that might be most effective for each case.

Previous studies have found that the stages of change with the highest prevalence are the maintenance and preparation stages (Mullan and Markland, 1997; Marshall and Biddle, 2001; Daley and Duda, 2006; Thogersen-Ntoumani and Ntoumanis, 2006). Studies on the Mexican population that have analyzed this prevalence differ from the aforementioned findings because most of them agree that contemplation is the most prevalent stage, suggesting cross-cultural differences (Zamarripa et al., 2018). Thus, interest has been shown in examining the applicability of psychological theories in different countries and cultures (e.g., Duda and Hayashi, 1998; Gangyan and Hing-chu, 2007).

From a theoretical perspective, it is necessary to have measures with rigorous psychometric properties within and across countries that can facilitate cross-cultural comparative research and help to understand cultural variations in the motivation to exercise in the specific Mexican context and around the world. Moreover, examining gender invariance will provide further support for testing motivational regulations by gender. For example, previous studies have reported gender differences in levels of motivational regulations (e.g., Daley and Duda, 2006; Johnson et al., 2011; Su et al., 2015), while in other studies no gender differences were observed (e.g., Lutz et al., 2003; Shen et al., 2008). To clarify these inconsistencies, Guérin et al. (2012) conducted a meta-analysis with 27 studies to examine gender-based mean differences in motivational regulations for exercise. Results were in line with findings demonstrating no gender differences on motivational regulations. However, not all studies looked at gender invariance of the instruments used for the study. So, it is important to analyse measurement invariance for further research to clarify the gender differences in motivational regulations. In addition, from a practical perspective, these instruments will be useful in evaluations prior to any type of intervention designed to promote physical exercise because they can identify the person's motivational level and his/her predisposition to change. This information would make it possible to direct the intervention in a more suitable way, in order to reduce the high rates of physical inactivity and obesity in the Mexican population (e.g., Zamarripa et al., 2013; Dávila-Torres et al., 2015).

Therefore, the aim of this study was twofold: (1) to translate the original English version of the BREQ-3 (Markland and Tobin, 2004; Wilson et al., 2006) into Mexican Spanish and examine its factor structure (six dimensions), reliability, and discriminant validity. Given that comparisons across genders are meaningful only when gender invariance has been empirically demonstrated, multi-group confirmatory factor analyses will be performed to test invariance by gender; and (2) to analyse variations in the six regulations across the stages of change in a sample of individuals who live in the metropolitan area of the city of Monterrey, Nuevo Leon, Mexico. Based on the theoretical propositions (SDT and stages of change model) and previous work (Mullan and Markland, 1997; Daley and Duda, 2006; Thogersen-Ntoumani and Ntoumanis, 2006), it is hypothesized that participants who report more self-determined exercise regulations will indicate being in later stages of change than those who endorse less self-determined regulations, which would indicate being in the earlier stages of change. Due to the prevailing obesity in Mexico, it is expected that the majority of the participants will be in the stages of pre-contemplation, contemplation, and preparation; that is, subjects will be inactive or active, but they will not comply with the recommendations for healthy practice.

## Materials and Methods

### Participants

Participants were 530 individuals (40.2% men and 51.8% women; *M*_age_ = 32.22; *SD* = 15.27; range = 11 – 76 years; median = 30.00 years), who lived in the metropolitan area of Monterrey, Nuevo Leon (Mexico) and were recruited via convenience sampling. Most of the sample were < 30 years old (49.8%), followed by 30 to 44 years (24.2%), 45 to 59 years (18.4%), and 60 years or older (7.6%).

### Instruments

The BREQ-3 is a 23-item questionnaire divided into six subscales: intrinsic regulation (four items; e.g., “I exercise because it's fun”), integrated regulation (four items; e.g., “I exercise because it is consistent with my life goals”), identified regulation (three items; e.g., “I value the benefits of exercise”), introjected regulation (four items; “I feel guilty when I don't exercise”), external regulation (four items; e.g., “…because other people say I should”), and amotivation (four items; e.g., I don't see why I should have to exercise”). The questionnaire begins with the stem “I do physical exercise…” Responses are given on a 5-point scale ranging from 0 (definitely not true) to 4 (definitely true). Previous research has confirmed the reliability of this instrument (Wilson et al., 2006; González-Cutre et al., 2010).

To find out what stage of change the person was in, we used a Mexican-Spanish version (Zamarripa et al., 2018) of the stages of change questionnaire for physical activity by Marcus and Forsyth (2009). First, we asked the participants to read the following heading: “*physical activity or exercise includes activities such as walking quickly, running, riding a bicycle, swimming, or any other activity in which exercise is at least as intense as these activities”*. Then we asked them to indicate yes or no in response to the following statements: (1) I am currently physically active; and (2) I intend to be more physically active in the next 6 months. Participants who answered yes to question (1) did not answer question (2) and went on to read the following heading: “*For activity to be regular, you must do a total of 30 min or more per day at least 5 days a week. For example, you could walk once for 30 min or do three 10-min sessions, for a daily total of 30 min.”* Afterwards, we asked the participant to answer yes or no to the following statements: (3) I currently do regular physical activity; and (4) I have been doing regular physical activity for the past 6 months. The participants were placed in one of the five stages according to the algorithm presented in Table 1. Previous research has confirmed the reliability of this instrument (Marcus and Forsyth, 2009; Zamarripa et al., 2018). Furthermore, in an independent evaluation (Zamarripa et al., 2018), the criterion validity of the measure was supported through its relationship with self-reported physical activity in a Mexican sample (*p* < 0.001).

**Table 1:** Algorithm for the categorization and distribution of the sample in stages of change.

	**Statement number**			
	**(1)**	**(2)**	**(3)**	**(4)**	**Stage**	***f***	**%**
If they responded	No	No	—–	—–	Precontemplation	108	20.4
If they responded	No	Yes	—–	—–	Contemplation	120	22.6
If they responded	Yes	—–	No	—–	Preparation	69	13.0
If they responded	Yes	—–	Yes	No	Action	68	12.8
If they responded	Yes	—–	Yes	Yes	Maintenance	165	31.1

*f, frequency; %, percentage*.

### Procedure

The instrument was administered through personal interviews, with previous consensus, by trained interviewers in the home of the interviewee, who was randomly selected. The interviewer recorded the interviewee's responses. All the participants were informed of the study's objective and voluntary nature. They were told that their answers and data management would be completely confidential, and that there were no right or wrong answers. They were also asked to respond with sincerity and honesty.

The English version of the BREQ-3 was translated into Mexican Spanish using the translation-back translation procedure (Hambleton and Kanjee, 1995). The translation was carried out by a professional translation service contracted by the study investigators. A group of six experts was formed, consisting of three specialists with doctorates who work in the area of physical activity and sport psychology, two specialists with sufficient experience in the validation of psychological instruments, and a translator specialized in the area of physical activity and sports, in order to discuss the discrepancies in the translation and obtain the first version of the instrument in Mexican Spanish. This version of the questionnaire was again translated into English by a different professional translation service, and the two versions of the instrument were compared: the original and the translation. The differences in the versions were again analyzed, and necessary changes were made to facilitate the understanding of the items, thus achieving the final version of each of the scales. Table 2 shows the items that make up each of the six subscales.

**Table 2:** Descriptive statistics for the behavioral regulation in exercise questionnaire (BREQ-3) items.

**Yo hago ejercicio f**í**sico…[I do physical exercise…]**	
**No**.	**Items**	**Mean**	**SD**	**Skewness**	**Kurtosis**
**INTRINSIC REGULATION**					
04	…porque creo que el ejercicio es divertido. […I exercise because it's fun.]	2.52	1.22	−0.43	−0.81
12	…porque disfruto con las sesiones de ejercicio. […I enjoy my exercise sessions.]	2.45	1.30	−0.40	−0.98
18	…porque encuentro el ejercicio una actividad agradable. […I find exercise a pleasurable activity.]	2.65	1.24	−0.59	−0.67
22	…porque hacer ejercicio me resulta placentero y satisfactorio. […I get pleasure/satisfaction from exercise.]	2.50	1.27	−0.44	−0.85
**INTEGRATED REGULATION**					
05	…porque concuerda con mi forma de vida. […I exercise because it is consistent with my life goals.]	2.31	1.28	−0.23	−1.01
10	…porque considero que el ejercicio físico forma parte de mí ser. […I consider exercise to be part of my identity.]	2.23	1.26	−0.17	−1.00
15	…porque veo el ejercicio físico como una parte fundamental de lo que soy. […I consider exercise a fundamental part of who I am.]	2.34	1.27	−0.27	−0.98
20	…porque considero que el ejercicio físico concuerda con mis valores. [I consider exercise consistent with my values.]	2.19	1.21	−0.20	−0.84
**IDENTIFIED REGULATION**					
03	…porque valoro los beneficios que tiene el ejercicio físico. […I value the benefits of exercise.]	2.78	1.25	−0.71	–0.58
09	…porque para mí es importante hacer ejercicio regularmente. […It's important to me to exercise regularly.]	2.67	1.23	−0.55	−0.75
17	…porque pienso que es importante hacer el esfuerzo de ejercitarme regularmente. […It's important to make an effort to exercise.]	2.66	1.24	−0.58	−0.67
**INTROJECTED REGULATION**					
02	…porque me siento culpable cuando no lo hago. […I feel guilty when I don't exercise.]	1.58	1.36	0.32	−1.14
08	…porque me siento avergonzado si falto a la sesión. [I feel ashamed when I miss exercise.]	1.44	1.32	0.41	−1.02
16	…porque siento que he fallado cuando dejo de hacer ejercicio por un tiempo. […I feel like a failure when I haven't exercised.]	2.03	1.35	−0.08	−1.14
21	…porque me pongo nervioso si no hago ejercicio regularmente. […I get restless if I don't exercise regularly.]	1.39	1.35	0.54	−0.97
**EXTERNAL REGULATION**					
01	…porque los demás me dicen que debo hacerlo […because other people say I should.]	1.26	1.29	0.66	−0.74
07	…porque mis amigos/familia/pareja me dicen que debo hacerlo. […because my family/friends/partner say I should.]	1.45	1.36	0.44	−1.12
13	…porque otras personas se enojarían conmigo si no hago ejercicio. […Because others will not be pleased with me.]	1.16	1.30	0.73	−0.79
19	…porque me siento presionado por mis amigos/familia/pareja para realizar ejercicio [I feel under pressure from others.]	1.28	1.31	0.57	−0.94
**AMOTIVATION**					
06	…pero no sé por qué tengo que hacerlo. […I don't see why I should have to exercise.]	1.30	1.32	0.57	−0.97
11	…pero no sé por qué me molesto en hacer ejercicio físico. […I can't see why I should bother exercising.]	1.28	1.29	0.59	−0.86
14	…pero no veo el sentido de hacer ejercicio. […I don't see the point in exercising.]	1.20	1.31	0.67	−0.85
23	…pero pienso que hacer ejercicio es una pérdida de tiempo. […I think exercising is a waste of time.]	1.15	1.38	0.79	−0.81

*No, number; M, mean; SD, standard deviation*.

All the subjects gave written informed consent in accordance with the Declaration of Helsinki. The present research was conducted in accordance with international ethical guidelines, which are consistent with American Psychological Association (APA) guidelines. Ethical approval for the study was obtained from the Universidad Autonoma de Nuevo Leon (Mexico) ethics review committee (No. 16CI19039021).

### Statistical Analysis

Before proceeding on to data analysis, the data set was screened for multivariate outliers, and the normality of the distributions was also examined. Missing data were very small (< 0.1%) so we chose not to replace these scores. According to Tabachnick and Fidell (2007), when < 5% of data points are missing from the data set at random, almost any method for handling missing values yields similar results.

BREQ-3 items were subjected to confirmatory factorial analysis (CFA) using LISREL 8.80 (Jöreskog and Sörbom, 2006) to test whether the six-factor structure adequately fit the data collected from a Mexican population. Considering the ordinal nature of the data, the sample size, the number of response categories (k = 5), and the fact that the items were normally distributed (see Table 2), maximum likelihood was used to estimate model parameters, and the polychoric correlation matrix and the asymptotic covariance matrix were used as input for the analyses. We considered CFI, NNFI, and RMSEA to evaluate goodness of fit and the parameter estimates. CFI and NNFI values >0.95 indicate an acceptable fit (Hu and Bentler, 1999). For RMSEA, values less than or equal to.08 are considered satisfactory (Cole and Maxwell, 1985).

A multi-sample CFA was employed to examine whether the BREQ-3 displayed invariance by gender. Incremental fit indices were estimated to compare the alternative models' goodness of fit. A difference of 0.01 or less between values of CFI (Cheung and Rensvold, 2002) and NNFI (Widaman, 1985) reflect practically irrelevant differences between models. Regarding RMSEA, an increase of 0.015 or less between alternative models indicates irrelevant differences (Chen, 2007) and therefore the most parsimonious model should be selected.

Internal consistency was evaluated using Cronbach's (1951) alpha, Composite Reliability (CR), and the Average Variance Extracted (AVE). Alpha and CR values should be above 0.70 to be considered acceptable. AVE values of 0.50 and greater are considered acceptable (Fornell and Larcker, 1981). Convergent validity was analyzed considering that the items load high on their respective constructs, and considering values of AVE ≥0.50. Discriminant validity was assessed using the Fornell and Larcker (1981) criterion, that is, the square root of each construct's AVE should have a greater value than the correlations with other latent constructs. Another measure for discriminant validity has been to examine the correlation between the constructs that should be lower than 0.85 (Kline, 2005).

Variations in the different regulations across the stages of change were analyzed using multivariate analysis of variance (MANOVA) and *post hoc* Tukey tests. A value of partial eta-squared above 0.06 is considered a moderate effect size, and a value above 0.14 is considered a large effect size (Cohen, 1988). For these analyses, we used the SPSS 21 program.

## Results

### Confirmatory Factorial Analysis

**Figure 1:**
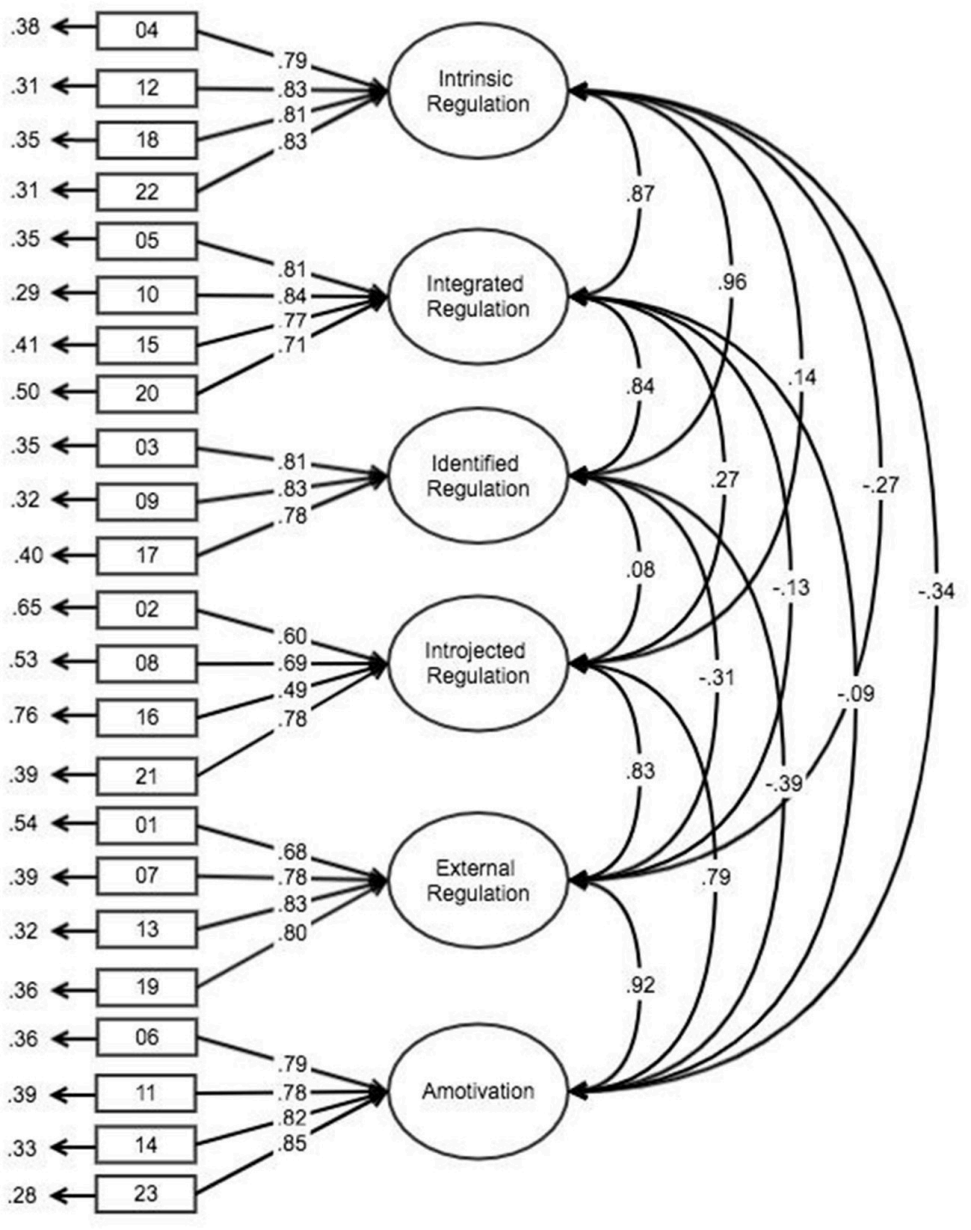
Standardized parameters (factorial weights, covariance factors, and measurement errors) for the BREQ-3. All factor loadings were significant (*p* < 0.05).

The proposed factorial structure adequately fitted the data (χ^2^ (215) = 548.93, *p* < 0.01; NNFI = 0.980, CFI = 0.983, RMSEA = 0.0545), confirming the validity of the six-factor model. Factor loadings of the model ranged from 0.49 to 0.85 and loaded significantly in their respective constructs (Figure 1) supporting measurement convergent validity. Means, standard deviations, skewness and kurtosis of BREQ-3 items are displayed in Table 2, demonstrating acceptable normality of score distribution.

### Measurement Invariance

Table 3 shows that the model was invariant across gender. The CFA results showed that the proposed factorial structure was acceptable for males and females, confirming the validity of the six-factor model in the two groups considered separately (Models M0a and M0b). Model 1 (configural invariance), where no equality constrains were imposed, showed a good fit to the data, and so this model was used as the baseline model. Results of Model 2 (metric invariance), Model 3 (scale invariance), and Model 4 (latent means invariance) showed an acceptable fit for gender invariance. Compared to the baseline model (M1), the increase in NNFI, and CFI did not exceed the criterion value of 0.01, and the increase in RMSEA did not exceed the criterion value of 0.15.

**Table 3:** Goodness of fit indices of gender invariance models.

**Model**	**Model description**	**χ^2^**	**df**	**RMSEA**	**NNFI**	**CFI**	**ΔRMSEA**	**ΔNNFI**	**ΔCFI**
M0a	Baseline model male sample	260.548^*^	215	0.0518	0.984	0.987		
M0b	Baseline model female sample	409.433^*^	215	0.0580	0.973	0.977		
M1	Configural invariance	859.081^*^	468	0.0566	0.978	0.980		
M2	Metric invariance	955.923^*^	491	0.0603	0.975	0.976	0.004	0.003	0.004
M3	Scale invariance	1038.515^*^	502	0.0641	0.972	0.972	0.007	0.006	0.008
M4	Latent means invariance	1109.987^*^	470	0.0716	0.969	0.970	0.015	0.009	0.010

*df, degrees of freedom; RMSEA, root mean square error of approximation; NNFI, non-normed fit index; CFI, comparative fit index. All the Δ index comparisons are made with respect to the configural model (M1)*.

* *p < 0.01*.

### Descriptive Statistics

Table 1 shows the distribution of the participants in the different stages of change. The majority of the participants were located in maintenance (31.1%), followed by contemplation (22.6%), pre-contemplation (20.4%), preparation (13%), and action (12.8%). The total sample had higher scores on more autonomous regulations, with identified regulation standing out. By contrast, the regulation with the lowest score was amotivation, followed by external and introjected regulation (Table 4).

**Table 4:** Means, standard deviations, reliability, discriminant validity and bivariate correlations between the behavioral regulation in exercise questionnaire (BREQ-3) subscales.

**Variable**	**Alpha**	**CR**	**AVE**	**Mean**	**SD**	**1**	**2**	**3**	**4**	**5**	**6**
1.Intrinsic regulation	0.84	0.89	0.67	2.53	1.03	1	0.75	0.92	0.02	0.07	0.12
2.Integrated regulation	0.83	0.86	0.62	2.27	1.02	0.69^**^	1	0.70	0.07	0.02	0.01
3.Identified regulation	0.78	0.85	0.65	2.70	1.03	0.76^**^	0.67^**^	1	0.01	0.09	0.15
4.Introjected regulation	0.68	0.74	0.42	1.61	0.96	0.19^**^	0.23^**^	0.11^*^	1	0.68	0.62
5.External regulation	0.81	0.86	0.60	1.29	1.05	−0.20^**^	−0.09^*^	−0.23^**^	0.57^**^	1	0.84
6.Amotivation	0.83	0.88	0.66	1.23	1.08	−0.23^**^	−0.07	−0.30^**^	0.52^**^	0.73^**^	1

*CR, composite reliability; AVE, average variance extracted; SD, standard deviations. Above the diagonal are the values of inter-construct correlations*.

** *p < 0.01*,

* *p < 0.05*.

### Internal Consistency, Convergent and Discriminant Validity

Table 4 shows the reliability results. The alpha reliabilities ranged from 0.68 to 0.84, which is considered adequate. The composite reliabilities ranged from 0.74 to 0.89, which is considered satisfactory. In general, the AVEs of this study were above the recommended threshold of 0.50, ranged from 0.60 to 0.67, except for the value of the introjected regulation dimension, which exhibited a value of 0.42. The weak factorial load of some introjected regulation items results in an AVE below the recommended value of 0.50. These results support adequate convergent validity of the BREQ-3 scales.

The results of discriminant validity using the Fornell and Larcker (1981) criterion indicated a lack of discriminant between six of the 15 inter-construct dimensions (see Table 4), that is, between intrinsic regulation and integrated regulation, between intrinsic regulation and identified regulation, between integrated regulation and identified regulation, between introjected regulation and external regulation, between introjected regulation and amotivation, and between external regulation and amotivation. However, correlations among the subscales conformed to a simplex pattern, with stronger positive correlations between adjacent factors than with factors further away from the diagonal. Specifically, the results showed (Table 4) that the most autonomous regulations (intrinsic, integrated, and identified) had positive, moderate, and significant correlations with each other. In addition, these regulations also correlated positively but slightly with introjected regulation. By contrast, external regulation and amotivation correlated positively with each other and negatively with the more autonomous regulations. Following Kline's (2005) criteria, because inter-factor correlations are below 0.85, factor discrimination can be established among the questionnaire's dimensions. Overall, these results provide evidence of discriminant validity between the subscales of the BREQ.

### Multivariate Analysis of Variance

**Figure 2:**
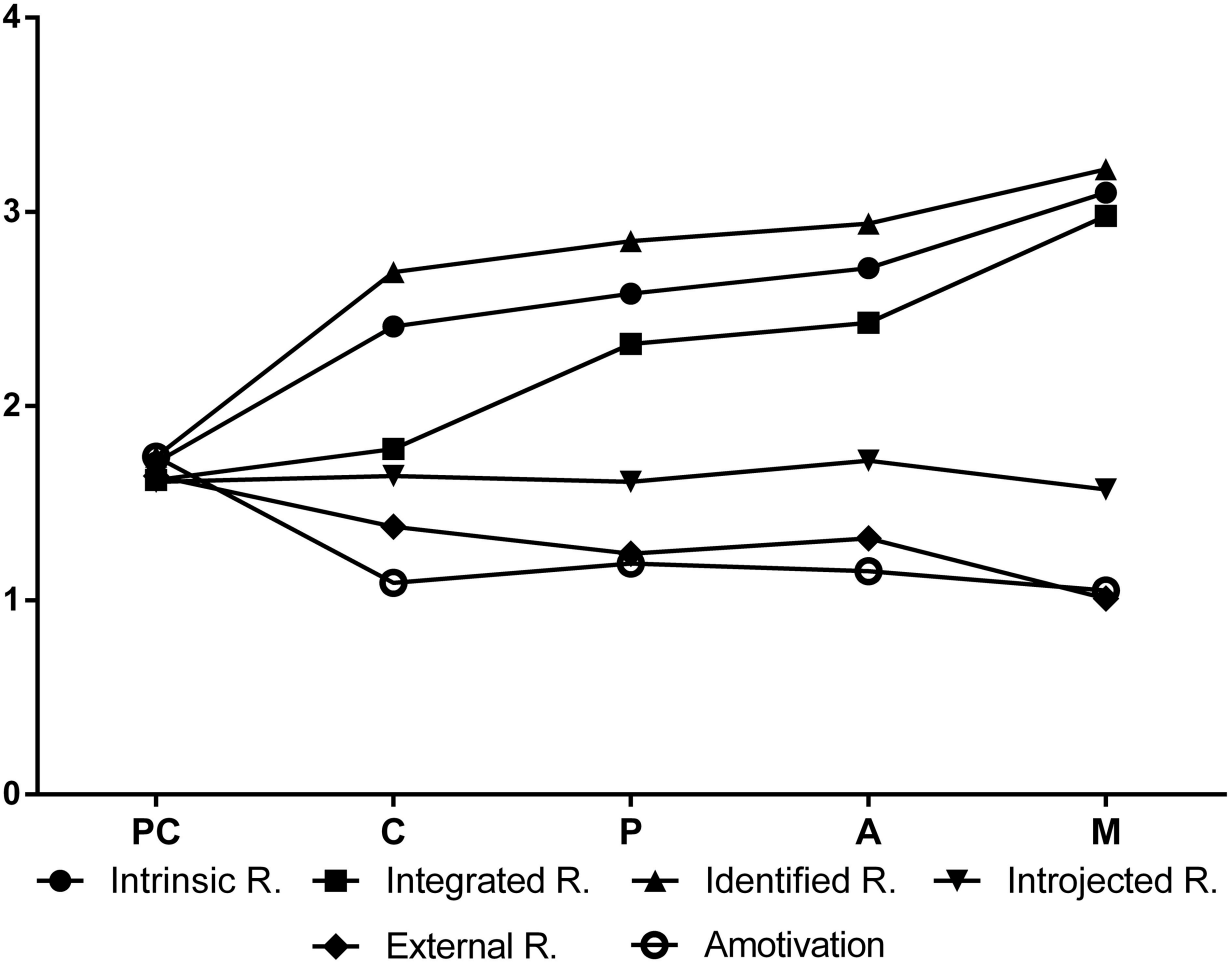
Differences among the stages of change in physical exercise regulation. PC = precontemplation; C = contemplation; P, preparation; A, action; M, maintenance; R, regulation.

The results of the MANOVA revealed statistically significant differences between the different stages of change and the forms of regulation (Wilks Lambda = 0.563, *F* = 13.47, *p* < 0.001, partial η^2^ = 0.13). Follow-up univariate analysis indicated significant differences in all the forms of regulation, except introjected regulation (see Table 5). Tukey's *post hoc* test revealed that participants in the pre-contemplation stage had significantly lower intrinsic regulation than those in all the other stages; by contrast, individuals in the maintenance stage showed significantly higher scores than those in all the other stages. The intrinsic regulation score increased from the pre-contemplation stage to the maintenance stage. With regard to integrated regulation, participants in the pre-contemplation and contemplation stages had lower scores than those in the preparation, action, and maintenance stages. In addition, participants in the maintenance stage had greater integrated regulation than individuals in the other stages. The integrated regulation scores also increased across the stages; however, there were no differences between pre-contemplation and contemplation, or between action and preparation. Regarding identified regulation, Tukey's *post hoc* test showed that participants in pre-contemplation had lower identified regulation than participants in all the other stages, whereas participants in maintenance presented higher identified regulation than those in all the other stages. As in the previous regulations, identified regulation also increased from the pre-contemplation stage to the maintenance stage; however, the differences between the stages of contemplation, preparation, and action were not significant (see Table 5; Figure 2).

**Table 5:** Means and standard deviations differences among the stages of change in physical exercise regulation.

	**Stages of change**				
	**Precontemplation**	**Contemplation**	**Preparation**	**Action**	**Maintenance**
**Variables**	***M* ±*SD***	***M* ±*SD***	***M* ±*SD***	***M* ±*SD***	***M* ±*SD***	***F*_[4, 523]_**	**Partial η^2^**
Intrinsic regulation	1.71 ± 0.84	2.41 ± 0.98	2.58 ± 0.91	2.71 ± 0.87	3.10 ± 0.87	40.46^*^	0.24
Integrated regulation	1.62 ± 0.78	1.78 ± 0.95	2.32 ± 1.00	2.43 ± 0.79	2.98 ± 0.79	53.73^*^	0.29
Identified regulation	1.74 ± 0.89	2.69 ± 0.91	2.85 ± 0.80	2.94 ± 0.90	3.22 ± 0.88	48.33^*^	0.27
Introjected regulation	1.61 ± 0.85	1.64 ± 0.09	1.61 ± 1.04	1.72 ± 0.95	1.57 ± 1.02	0.32	0.00
External regulation	1.64 ± 0.82	1.38 ± 1.08	1.24 ± 1.10	1.32 ± 1.10	1.01 ± 1.06	6.36^*^	0.05
Amotivation	1.74 ± 0.82	1.09 ± 1.02	1.19 ± 1.15	1.15 ± 1.10	1.05 ± 1.14	8.30^*^	0.06

* *p < 0.001*.

Unlike the previous regulations, the external regulation scores showed a decrease across the stages. Tukey's *post hoc* test revealed significant differences between the stages of pre-contemplation and maintenance, with participants in pre-contemplation showing greater external regulation than those in the maintenance stage. Finally, amotivation also showed significant differences across the different stages of change. Tukey's *post hoc* test showed that people in the pre-contemplation stage were more amotivated than those in the stages of preparation, action, contemplation, and maintenance (see Table 5; Figure 2).

## Discussion

The first objective of this study was to translate the English version of the Behavioral Regulation in Exercise Questionnaire-3 (BREQ-3), adapt it to Mexican Spanish, and analyse its factorial structure and internal consistency. The findings supported the factorial validity of the questionnaire. The six-factor model tested (intrinsic, integrated, identified, introjected, external, and amotivation) showed adequate fit indexes, and all the items presented factorial saturations above 0.40 in each of their factors; therefore, the full 23-item version was maintained. The internal consistency estimates for the six BREQ-3 subscales were adequate.

As Deci and Ryan (1985) predicted, the self-determination continuum emerged in the present sample. The correlations among the six subscales conformed to a simplex pattern; that is, adjacent factors were found to be more highly and positively correlated than with those at opposite ends of the continuum, thus supporting the construct validity of the BREQ-3. Furthermore, the correlation values between the factors were similar to those reported in previous studies with diverse samples and different language versions (e.g., Wilson et al., 2006; González-Cutre et al., 2010; Guedes and Sofiati, 2015; Cid et al., 2018). It should be noted that the correlations found in the present study ranged from low to moderate, indicating that the different factors correspond to constructs that are related, but independent from each other, offering support for the discriminant validity of the questionnaire. However, when using the Fornell and Larcker (1981) criterion, the results indicated a lack of discriminant especially among the closest regulations, i.e., between intrinsic, identified and integrated regulations; and between introjected regulation, external regulation and amotivation. This is consistent with results obtained in a meta-analysis testing the self-determination simplex pattern (Howard et al., 2017). This meta-analysis demonstrated that regulations can be ordered along the self-determination continuum and that it is possible to use all BREQ-3 regulation subscales individually. However, these authors supported the idea that it would be better to use a single motivation score representing degree of self-determination. According to Howard et al. (2017), future research might focused in how is the best way to operationalize motivational regulations (e.g., individual regulations, index of self-determination, more or less self-determined motivation).

The multi-sample CFA supported the measurement invariance of the BREQ-3 across gender groups, suggesting that participants responded in a similar fashion regardless of gender, allowing unbiased comparisons of average scores across male and female groups (Sass, 2011). In sum, this study is in line with previous studies that examined gender differences in motivational regulations (e.g., González-Cutre et al., 2010; Guedes and Sofiati, 2015; Su et al., 2015; Cid et al., 2018) indicating that the BREQ-3 in its Mexican version can be used to examine gender differences in exercise settings. To clarify the gender differences in motivational regulations is important not only for further research but also to identify which strategies are better to maximize people participation in exercise settings.

The second objective of the present study was to analyse the variations in the different regulations across the stages of change. As Daley and Duda (2006) pointed out, the transtheoretical model assumes a quantitative perspective, whereas SDT researchers are more concerned with the quality of motivation. Our results revealed that the quality of the motivation is related to the stages of change. That is, as people's motivation becomes more self-determined, their willingness to engage in physical exercise becomes more consistent. Thus, those located in the first stages of change, that is, where physical exercise is not done and there is no intention to do it, amotivation and external regulation predominated, compared to people who reported practicing physical exercise on a regular basis (action and maintenance). By contrast, intrinsic, integrated, and identified regulations were more predominant in individuals in the stages of action and maintenance, compared to those in the stages of contemplation and pre-contemplation.

All in all, these relationships between the stages of change and the forms of regulation provide evidence of the validity of the BREQ-3 based on its relationship with other variables. Specifically, participants who reported that they had exercised for 6 months or more (maintenance stage) had significantly higher scores on intrinsic regulation than participants who indicated that they did not exercise (precontemplation stage).

The predominance of less self-determined regulations in individuals in the early stages of change, as well as more self-determined regulations in individuals located in later stages, has also been found in previous studies (Mullan and Markland, 1997; Landry and Solmon, 2004; Daley and Duda, 2006; Thogersen-Ntoumani and Ntoumanis, 2006).

Our results confirmed the relation between the quality (motivational regulations) and quantity (stages of change) of the motivation to exercise in the Mexican population. Moreover, they confirm relationships found in other cultures using previous versions of the BREQ (Mullan and Markland, 1997; Landry and Solmon, 2004; Daley and Duda, 2006; Thogersen-Ntoumani and Ntoumanis, 2006).

Although previous studies found a higher prevalence of maintenance and preparation in the stages of change (Mullan and Markland, 1997; Marshall and Biddle, 2001; Daley and Duda, 2006; Thogersen-Ntoumani and Ntoumanis, 2006), and previous studies in the Mexican population found a prevalence of contemplation (Zamarripa et al., 2018), the prevalence in our sample is mainly of the maintenance, contemplation, and pre-contemplation stages. Daley and Duda (2006) combined the contemplation and pre-contemplation stages, arguing that these stages are similar with regard to attitudes and the intention to engage in future practice. With this in mind, our results can be polarized in two groups: a third (31.1%) of our sample that has actually engaged in exercise for more than 6 months (maintenance) and more than a third (20.4 and 22.6%) that is not interested at all in exercise (pre-contemplation and contemplation). These results have implications for interventions designed to keep people in the maintenance stage and help people in other stages to engage in physical activity.

The dynamic nature of stages of changes, that is, the fact that people can change stages as they perceive success or difficulties in making changes in their lifestyle (Prochaska et al., 1992b), offers opportunities for intervention. Our results suggest that the more self-determined a person's motivation is, the more he or she will do healthy exercise. According to Daley and Duda (2006), this has valuable implications for the design of physical activity interventions. Grounded in self-determination theory, providing autonomy support, that is, understanding what people want from physical activity and fostering opportunities for choice, enjoyment, and mastery, will increase the likelihood of people being in the maintenance stage.

## Conclusions

The Mexican version of the BREQ-3 is a reliable and valid instrument for studying motivational regulations in exercise contexts, and it can be used in future studies to increase the generation of knowledge and scientific production in this area in Mexico. Its factorial structure coincides with what has been found in previous studies, and it contributes to understanding adherence to and adoption of physical exercise by combining two theoretical proposals: self-determination theory (Deci and Ryan, 1985, 2002) and the stages of change from the transtheoretical model (Prochaska and Di Clemente, 1982; Prochaska et al., 1992a).

## Author Contributions

JZ and IC conceived the hypothesis of this study. JZ was responsible for ethical approval. RB, MD and OA participated in data collection. JZ and IC analyzed the data and drafted the article. All the authors contributed to data interpretation of the statistical analysis. All the authors read and approved the final version of the manuscript.

### Conflict of Interest Statement

The authors declare that the research was conducted in the absence of any commercial or financial relationships that could be construed as a potential conflict of interest.
